# Hemodynamic activity is not parsimoniously tuned to index-of-difficulty in movement with dual requirements on speed-accuracy

**DOI:** 10.3389/fnhum.2024.1398601

**Published:** 2024-07-09

**Authors:** Haibiao Ji, Zhi Chen, Yongjun Qiao, Jin Yan, Gaoxiang Chen, Qi Luo, Lijun Cui, Ya Zong, Qing Xie, Chuanxin M. Niu

**Affiliations:** ^1^Department of Rehabilitation Medicine, Ruijin Hospital, Shanghai Jiao Tong University School of Medicine, Shanghai, China; ^2^School of Medicine, Shanghai Jiao Tong University, Shanghai, China; ^3^School of Automotive and Mechanical Engineering, Changsha University of Science and Technology, Changsha, China

**Keywords:** functional near-infrared spectroscopy (fNIRS), cortical activation, reaching movement, Fitts' Law, index-of-difficulty

## Abstract

**Background:**

Reaching movements are crucial for daily living and rehabilitation, for which Fitts' Law describes a speed-accuracy trade-off that movement time increases with task difficulty. This study aims to investigate whether cortical activation in motor-related areas is directly linked to task difficulty as defined by Fitts' Law. Understanding this relationship provides a physiological basis for parameter selection in therapeutic exercises.

**Methods:**

Sixteen healthy subjects performed 2D reaching movements using a rehabilitation robot, with their cortical responses detected using functional near-infrared spectroscopy (fNIRS). Task difficulty was manipulated by varying target size and distance, resulting in 3 levels of index-of-difficulty (ID). Kinematic signals were recorded alongside cortical activity to assess the relationship among movement time, task difficulty, and cortical activation.

**Results:**

Our results showed that movement time increased with ID by 0.2974s/bit across all subjects (conditional r^2^ = 0.6434, *p* < 0.0001), and all subjects showed individual trends conforming Fitts' Law (all *p* < 0.001). Neither activation in BA4 nor in BA6 showed a significant correlation with ID (*p* > 0.05), while both the target size and distance, as well as the interaction between them, showed a significant relationship with BA4 or BA6 activation (all *p* < 0.05).

**Conclusion:**

This study found that although kinematic measures supported Fitts' Law, cortical activity in motor-related areas during reaching movements did not correlate directly with task difficulty as defined by Fitts' Law. Additional factors such as muscle activation may call for different cortical control even when difficulty was identical.

## 1 Introduction

Reaching movements are essential for daily living and clinical rehabilitation (Georgopoulos, 1986; Chang et al., 2008). These movements range from the simple act of interacting with touchscreens to complex upper-limb exercises in rehabilitation, such as button-pressing or nose-pointing (Konieczny et al., 2022). In reaching movements, a common observation is the *speed-accuracy trade-off* formulated by Fitts' Law (Fitts, 1954; Schmidt et al., 2018), which states that reaching movements become more challenging as distance increases and target-size decreases. Therefore, movement time grows proportionally in tasks with higher index-of-difficulty (ID). Using Fitts' Law, patient-machine interaction applications have been refined (Zimmerli et al., 2012), but the relationship between cortical activation and task requirements remains elusive. This gap obscures the choice of therapeutic parameters (e.g., dosage, repetition, and level of difficulty) in neurorehabilitation.

When assessing motor performance in patients with neurological impairments, Fitts' Law has been replicated in a wide range of tasks (MacKenzie, 1992; Bertucco and Sanger, 2014). Specifically, when patients with Parkinson's disease were instructed to move between two targets as quickly and accurately as possible, the movement time (MT) increased with index-of-difficulty (ID) conforming to Fitts' Law (Sakurada et al., 2018). In individuals with forearm amputation, the Fitts' mathematical relationship was also observed in grasping movements performed by myo-controlled prosthetic hands (Luo et al., 2021). In individuals who have suffered a stroke, the slope and intercept coefficients of Fitts' Law were associated with clinical assessments of motor impairment, as quantified by the Fugl-Meyer score (McCrea and Eng, 2005). In the design of patient-machine interfaces for children with dystonic cerebral palsy, the spacing and placement of touchscreen buttons were chosen based on index-of-difficulty, which improved upper extremity motor performance (Bertucco and Sanger, 2014). Overall, the correlation between motor performances (gauged by movement time) and task difficulty (gauged by index-of-difficulty) remained robust across contexts.

Nevertheless, the motor performance in reaching movements might not be explained solely by index-of-difficulty; alternatively, the performance could be affected separately by the distance and the size of the target. In movements with the same index-of-difficulty, trials with shorter distances exhibited characteristics such as increased variability, slower execution, and higher error rates in comparison to those over longer distances (Borish et al., 2020). If the increase of index-of-difficulty was achieved via smaller target size, this would result in reduced peak velocity, elongated deceleration, and a significant increase in movement time (Bohan et al., 2003); in contrast, if the increase in ID was achieved via longer distance, some studies reported no increase in the movement time (van Vliet and Sheridan, 2009). Other effects of increased target-size included larger accelerating torque as well as elevated agonist EMG during movement initiation (Corcos et al., 1989), whereas a decrease in distance resulted in unchanged rates of acceleration torque or agonist EMG (Gottlieb et al., 1989). In summary, although the index-of-difficulty could be altered via either the distance or the target-size, these two approaches may trigger different responses in the motor nervous system.

Inconsistent findings were also reported regarding whether cortical activation was mainly explained by index-of-difficulty. In reaching movements with high ID, both healthy individuals and stroke patients demonstrated intensified activation in Brodmann Areas 4 (mainly the primary motor cortex) (Barany et al., 2020; Revill et al., 2022). Additionally, healthy individuals exhibited increased activation in BA6 (mainly the supplementary motor cortex and premotor cortex) (Winstein et al., 1997; Seidler et al., 2004) detected using fMRI; on the contrary, other studies using the same movement paradigm did not elicit notable differences in BA4 (Buetefisch et al., 2014) or BA6 (Haar et al., 2017). Furthermore, when distance and target-size changed proportionally such that the index-of-difficulty remained unchanged, increased activation in BA6 was observed at shorter distances (Winstein et al., 1997). In clinical rehabilitation, motor therapy led to increased activation in contralateral BA4 and BA6 (Li et al., 2023), and this activation correlated with improvements in upper-limb functions (Johansen-Berg et al., 2002). Therefore, the unclear relationship between index-of-difficulty and motor performance may root in how cortical activity is modulated by task parameters.

In this study, we asked the question whether activity in motor-related cortices was tuned to index-of-difficulty in reaching movements that require both speed and accuracy. In large-extent movements such as reaching, it has not been easy to establish the association between cortical activity and motor performances, mainly due to the lack of imaging techniques insusceptible to head movements. To this end, functional near-infrared spectroscopy (fNIRS) makes it suitable for the detection of cortical responses, especially during large-extent movements that may drag the head or trunk, as has been shown in previous studies (Hou et al., 2021). Here, healthy subjects performed 2D reaching movements by holding the handle of a rehabilitation robot. The distances and target-sizes were changed such that each subject experienced 3 different IDs but 4 different combinations of distance and target-size. Hemodynamic and kinematic signals were recorded. We hypothesize that even though the kinematic measures might reconfirm Fitts' Law (i.e., the movement time correlates with ID), it is unlikely that the activity in motor-related cortices correlates parsimoniously with ID.

## 2 Methods

### 2.1 Participants

Sixteen healthy participants [14 male, 2 female; mean age = 59.19 ± 3.80 years; all right-handed according to the Edinburgh Handedness Inventory (Oldfield, 1971)] volunteered in this study. Subject recruitment and experimentation were completed from October 2023 to January 2024 at the Department of Rehabilitation Medicine of Ruijin Hospital affiliated with Shanghai Jiao Tong University School of Medicine, with a total of 18 subjects recruited. The inclusion criteria for the experiment were: (1) Healthy volunteers without neurological or musculoskeletal diseases; (2) No psychiatric diseases; (3) Age between 50–65 years old. The exclusion criteria were: (1) Reported fatiguing or sleep deprivation within 2 days; (2) Unqualified fNIRS signal quality as reported by the device. Written informed consent was obtained from each participant following approval from the Ethics Committee of Ruijin Hospital, School of Medicine, Shanghai Jiao Tong University (No. 209 of 2020).

### 2.2 Task design

**Figure 1:**
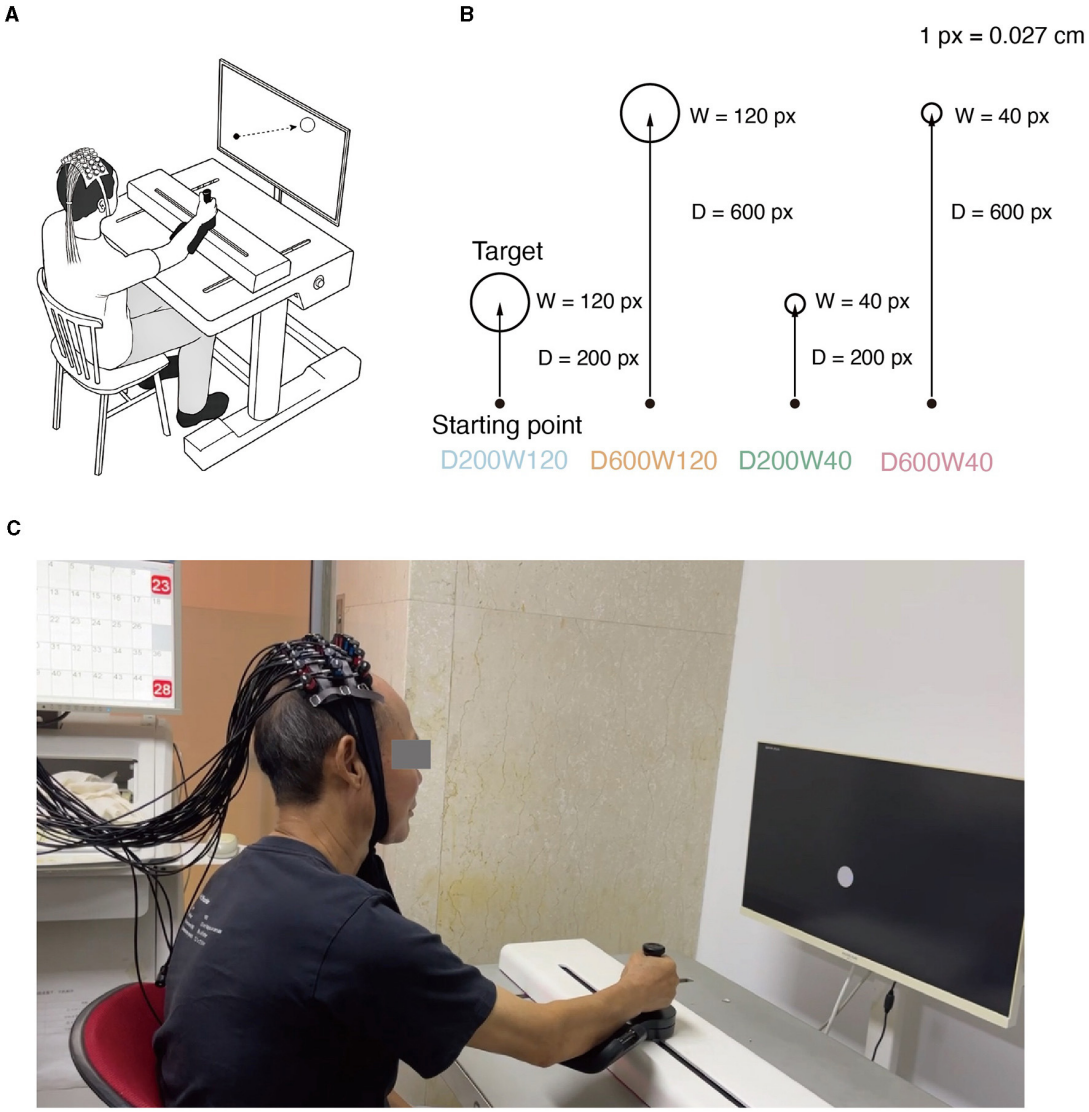
**(A)** Experimental setup. While manipulating the M2 Pro handle, the subject experiences real-time visualization of the starting point's movement on the screen. Meanwhile, fNIRS monitors the physiological activity within the brain's ROI (Region of Interest) of the subject. **(B)** Reaching movement tasks with varying difficulty levels. Four distinct conditions emerge through the combination of two different target distances and widths. **(C)** Actual scenes of the experiment.

The experiment was conducted in a quiet room by a physical therapist. Participants sat in an armless chair, facing a computer screen set at an appropriate distance and height for the collection of fNIRS data (Figures 1A, C). The upper-limb rehabilitation robot (ArmMotus™ M2 Pro, Fourier Intelligence Co., Ltd., Shanghai, China) provided the kinematic and kinect environment required in the study. During the experiment, a 28-inch screen with a resolution of 1,920 × 1,080 was placed in front of the participants, and the screen size was proportional with the working space of the rehabilitation robot (52 cm × 29.25 cm). On the screen, 1 pixel corresponded to 0.027 cm on the workspace of the robot. The handle of the robot provided a simulated mass of 10 kg and a simulated damping of 80N·s/m. The center of the handle was illustrated on the screen as a circle with a diameter of 20 pixels. The scheduling of experimental parameters and their coordination with devices were administered by a custom-written software XO (v1.42).

**Figure 2:**
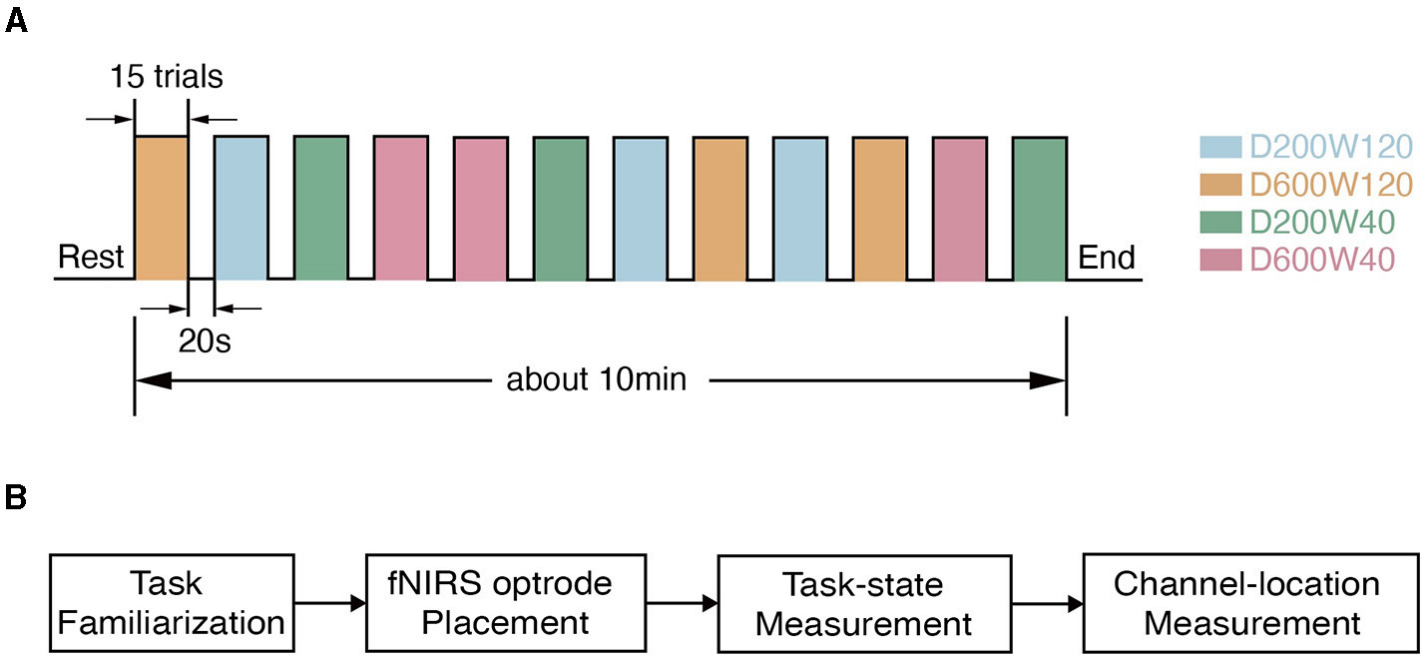
Experimental procedure. **(A)** The timeline during data collection. **(B)** The flow chart of the experiment within one visit. Each subject went through task familiarization (about 10 min), fNIRS optrode placement (about 5 min), and task-state measurement (about 10 min), and channel-location measurement (about 5 min).

The reaching movement was performed under 4 different conditions (Figure 1B), which were constructed by varying two variables: distance (D = 200 or 600 pixels) and size/width (W = 40 or 120 pixels). The four conditions corresponded to three different IDs: D200W120 represented the lowest task difficulty (ID = 1.74 bit); D600W120 and D200W40 represented moderate task difficulties (ID = 3.32 bit); D600W40 represented the highest task difficulty (ID = 4.9 bit). The experimental task comprised 12 blocks, containing 4 different conditions repeated 3 times in a random order. Each block included 15 trials of the same condition. Completion of the entire set of tasks took approximately 10 min (Figure 2A). The mean durations for each block in each condition are as follows (mean ± SD):

D200W120: 20.07 ± 1.32s;

D600W120: 26.35 ± 1.92s;

D200W40: 27.10 ± 1.57s;

D600W40: 30.54 ± 0.77s.

At the onset of the experiment, participants were instructed to maintain an upright sitting position and place their left hands on their laps. The right hand was strapped to the robotic handle, with a cast supporting the forearm. At the beginning, the starting point was displayed at the bottom right corner of the screen. Participants were asked to move the handle from the starting point to the target as quickly as possible in each trial within 2 seconds, during which the target faded out until invisible. Upon completion of a trial, the next target would appear immediately, and subjects would then move from the current position toward the target. Once a block (15 trials of reaching) was completed, the handle was returned to the bottom right corner, followed by a 20-second resting interval, signaling readiness for the next block.

The multichannel fNIRS device (model ETG-4100, HITACHI Inc., Japan) captured brain-hemodynamic signals at a 10 Hz sampling rate. The device measured the absorption of near-infrared light at two wavelengths (695 nm and 830 nm) and computed the corresponding hemoglobin and deoxyhemoglobin density in accordance with the modified Beer–Lambert Law (Delpy et al., 1988). Regions of interest (ROIs) included contralateral BA4 (mainly M1) and BA6 (encompassing SMA and PMC), specifically because BA4 had been shown to be related to the execution of movement and BA6 to higher order motor functions (Cheney, 1985). The selection of ROIs was also significant with previous studies (Chen et al., 2022, 2023).

Optodes were positioned on the head using a high-density 3 × 10 probe with 16 emitters and 14 detectors, forming 44 channels that covered the BA4 and BA6 of both hemispheres, similar to previous studies (Chen et al., 2022, 2023). The probe placement followed the international 10–20 system (Homan, 1988) to ensure coverage of the ROIs. The midpoint between channels 22 and 23 was first aligned with the Cz point, then the front brim of the probe was set parallel with the coronal plane of the skull. The location of each channel was measured using a 3D magnetic-space digitizer (Polhemus Patriot, Polhemus Inc., Vermont, USA). The MNI coordinates of each electrode were calculated using NFRI functions (Singh et al., 2005). The anatomic labeling and the corresponding probabilistic registration of a representative subject is shown in Table 1. The flow of the experiment is depicted in Figure 2B.

**Table 1:** Location of channels for subject 04.

**Channel**	**Anatomical label in BA**	**MNI coordinate**	**Probability**
14	4—primary motor cortex	(−6.33, −35.33, 79)	0.756
15	4—primary motor cortex	(−17.67, −35.67, 78)	0.566
16	4—primary motor cortex	(−31, −35, 73.67)	0.48
24	6—pre-motor and supplementary motor cortex	(−16.67, −16.67, 78)	0.723
25	6—pre-motor and supplementary motor cortex	(−29.67, −18.33, 74)	0.681
26	4—primary motor cortex	(−43.333, 19.333, 66.667)	0.642
32	6—pre-motor and supplementary motor cortex	(−5.67, −4.67, 75.33)	1
33	6—pre-motor and supplementary motor cortex	(−15.67, −3.33, 75.33)	1
34	6—pre-motor and supplementary motor cortex	(−29.33, −3.67, 69.33)	1
35	6—pre-motor and supplementary motor cortex	(−41.67, −2.67, 62.33)	0.912
36	6—pre-motor and supplementary motor cortex	(−54, −3.33, 52.67)	0.825

### 2.3 Kinematic data acquisition and processing

**Figure 3:**
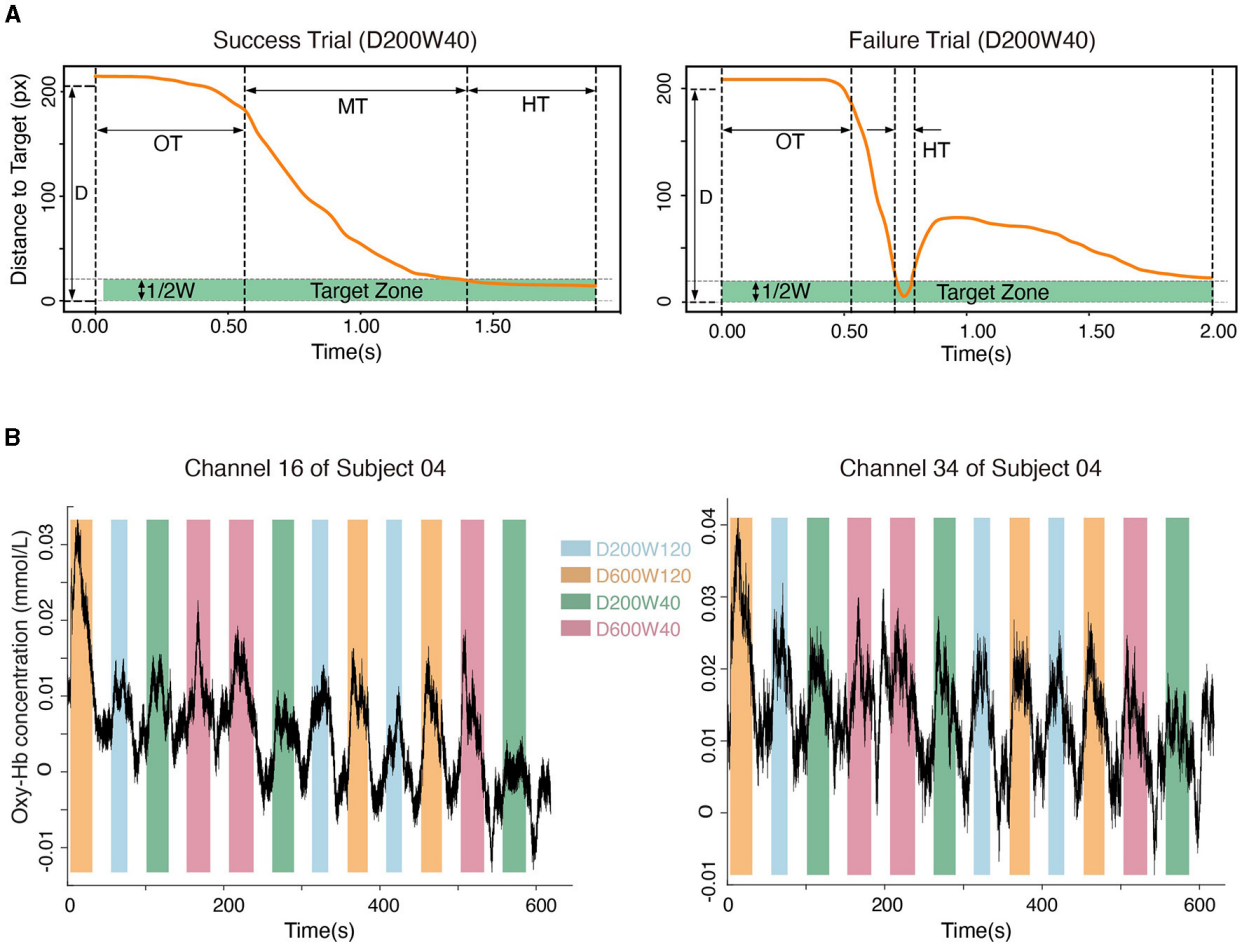
**(A)** The movement process in a typical success trial and a failure trial. “OT” represents the time leaving the starting point, “MT” represents the duration of moving from the starting point to the target, and “HT” represents the time holding in the target zone. **(B)** Hemodynamic signals from Channel 16 (located in BA4) and Channel 34 (located in BA6). Data from subject 04 are shown.

The position and state of the cursor were recorded at a sampling rate of 60 Hz using the robot. Success trials meant that the cursor entered the target zone within 2s from the target appearance and remained inside for 0.5s (HT = 0.5s); Failure trials meant that the cursor did not meet the criteria of success within 2 seconds (Figure 3A). The movement time (MT) was recorded for Success Trials only.

Overshoot occurred when a trial reached the target but exited within 0.5 seconds. The percentage of overshoot was calculated by dividing the count of overshot trials by the total number of trials. Throughput (TP) was another measure for the rate of information transmission, defined as follows:


(1) $$\begin{array}{l} \begin{array}{c} \text{T}\text{P}=\frac{1}{\text{N}}\overset{\text{N}}{\underset{\text{i}=1}{\sum }}{\text{I}\text{D}}_{\text{i}}/{\text{M}\text{T}}_{\text{i}} \end{array} \end{array}$$

where *N* is the number of all successful trials (Equation 1).

### 2.4 Brain hemodynamic data acquisition and processing

Oxy-Hb signals were used to quantify cortical activity in this study because they were sensitive to regional-cerebral-blood-flow fluctuations (Jalalvandi et al., 2021). The hemodynamic signals for each channel within the ROIs were recorded (Figure 3B). The pre-processing of oxy-Hb consisted of 3 steps: (1) the raw oxy-Hb signals were detrended using the linear-detrending method; (2) motion artifacts were removed using temporal derivative distribution repair (TDDR); (3) the oxy-Hb signals were band-pass filtered with cut-off frequencies at 0.01 Hz and 0.08 Hz (third-order Butterworth) to remove physiological artifacts (such as heartbeats, breath, and Mayer wave) and high-frequency noise. Cortical activation was calculated by subtracting the averaged signals during the 5 seconds preceding the onset of each block from the oxy-Hb concentration during the task phase of each block (Nishiyori et al., 2016). The general linear model (GLM) approach (Friston et al., 1994) was used to calculate the blood response index (the Beta coefficient) of each channel in each condition. Beta coefficients, which indicated both the direction (positive or negative) and magnitude of oxy-Hb changes for each condition, were calculated for every subject and channel (Hou et al., 2021). Channels were excluded from analyses if their coefficient of variation exceeded 30% (Davison et al., 2015), and < 1.1% of the total channels were excluded. The NIRS-KIT toolbox (version 2.0) (Hou et al., 2021) was used for hemodynamic signal processing.

To understand the ROI-based activation by taking advantage of the 3D magnetic-space digitization, we calculated a weight-adjusted activation by discounting how much the top 3 channels are likely to be registered to the ROI (Okamoto et al., 2009). The weight-adjusted hemodynamic signal for each ROI was:


(2) $$\begin{array}{l} \begin{array}{c} {\text{o}xyH\text{b}}_{\text{R}O\text{I}}=\frac{\overset{3}{\underset{\text{i}=1}{\sum }}{\text{P}}_{\text{i}}\;\cdot \;{\text{o}x\text{y}}_{\text{i}}}{\overset{3}{\underset{\text{i}=1}{\sum }}{\text{P}}_{\text{i}}} \end{array} \end{array}$$

where *oxyHb*_*ROI*_ represented the beta value of oxy-Hb signals for each ROI (BA4 and BA6, Equation 2), and *P*_*i*_ represented the most likelihood of channel i out of three, ranked by the likelihood in the probabilistic registration, displayed in Table 1. The MNI coordinates for the weighted channels of all participants are provided in the Supplementary material.

### 2.5 Statistical analysis

In this study, A linear regression model was fitted to examine the relationship between ID and MT to determine if the data conformed Fitts' Law. The performance of each participant was first evaluated by whether a trial was likely to be a success or failure. We employed a Generalized Linear Mixed-effect Model (GLMM) with a binary logistic regression approach to evaluate the effects of distance (D) and width (W), as well as their interaction (D × W), on the probability of success:


(3) $$\begin{array}{l} \begin{array}{c} \text{S}ucces\text{s}\;\text{p}robabilit\text{y}\;\sim \;\text{D}+\text{W}+\text{D}\times \text{W}+\left(1|\text{s}ubjec\text{t}\right) \end{array} \end{array}$$

where the term (1|*subject*) accounted for subject-specific intercepts as a random effect (Equation 3).

GLMM was also applied to assess the impact of ID on the success probability (Equation 4):


(4) $$\begin{array}{l} \begin{array}{c} \text{S}ucces\text{s}\;\text{p}robabilit\text{y}\;\sim \;\text{I}\text{D}+\left(1|\text{s}ubjec\text{t}\right) \end{array} \end{array}$$

We conducted one-sample *t*-tests, each comparing changes in Oxy-Hb levels for every block from each channel against 0. To detect activations in BA4 and BA6, we conducted Wilcoxon rank sum test due to non-normal distribution in the D200W120 and D600W120 conditions. Bonferroni corrections were also applied for multiple comparisons. The threshold for statistical significance was set at 0.05. A Linear Mixed-effects Model (LMM) was applied to analyze effects of ID on MT (Equation 5) and beta values (Equation 6). In LMM analyses, conditional r-square values were reported as the proportion of total variance explained through both fixed and random effects:


(5) $$\begin{array}{l} \text{M}\text{T}\;\sim \;\text{I}\text{D}+\left(1|\text{s}ubjec\text{t}\right) \end{array}$$

(6) $$\begin{array}{l} \text{B}et\text{a}\;\sim \;\text{I}\text{D}+\left(1|\text{s}ubjec\text{t}\right) \end{array}$$

LMM was also applied to analyze the effects of width and distance on MT (Equation 7) and beta values (Equation 8):


(7) $$\begin{array}{l} \text{M}\text{T}\;\sim \;\text{D}+\text{W}+\text{D}\times \text{W}+\left(1|\text{s}ubjec\text{t}\right) \end{array}$$

(8) $$\begin{array}{l} \text{B}\text{e}t\text{a}\;\sim \;\text{D}+\text{W}+\text{D}\times \text{W}+\left(1|\text{s}ubjec\text{t}\right) \end{array}$$

All statistical analyses were conducted using R (version 4.3.2). The GLMM and LMM were fitted using the “lme4” package (Bates, 2005), and the significances were calculated using the “lmerTest” package (Kuznetsova et al., 2017). The conditional r-squares were calculated using the “MuMIn” package (Bartoń, 2023). The pair-wise comparison of each condition on the probability of success were calculated using the “emmeans” package.^1^

## 3 Results

### 3.1 Kinematics analysis

**Figure 4:**
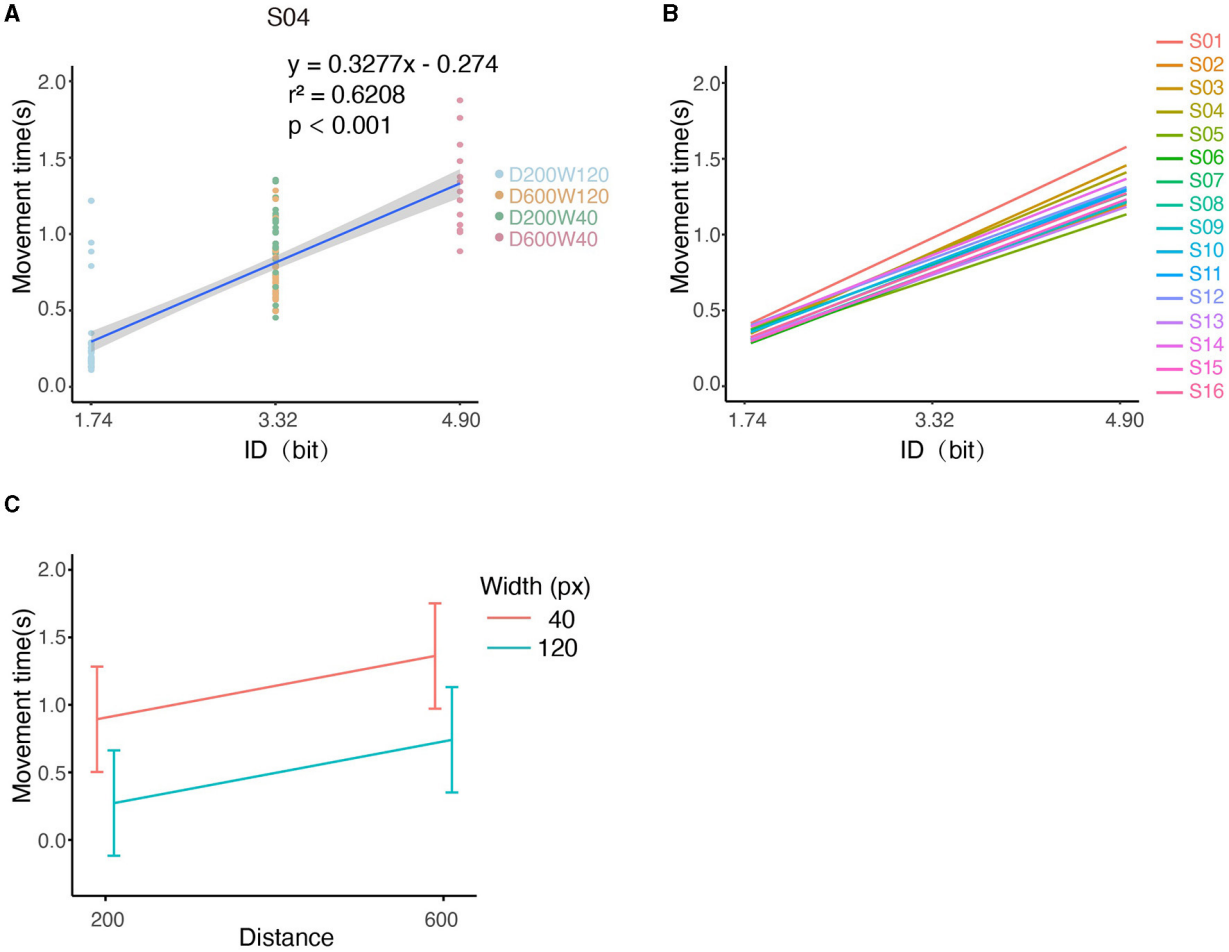
Illustration of kinematic performances across all conditions. **(A)** The relationship between MT and ID for S04. Note that tasks “D600W120” and “D200W40” had the same ID. **(B)** Relationship between MT and ID for each subject. **(C)** The relationship between the movement time and both distance and width.

We first analyzed whether there was a linear correlation between the Index of Difficulty (ID) and Movement Time (MT) according to Fitts' Law. For an individual subject (S04), the MTs and IDs of all trials are depicted in Figure 4A, and it can be seen that the MT increased with ID by 0.3277s/bit (r^2^ = 0.6208, *p* < 0.001), i.e., tasks with higher IDs are associated with longer completion time. For the pooled data from all subjects, MT was significantly increased with ID by 0.2974s/bit as shown by LMM (conditional r^2^ = 0.6434, *p* < 0.0001, Figure 4B). Analysis based on Equation 7 showed a significant effect of distance on movement time (slope = 0.117 ± 2.819 × 10^−3^, mean ± SE, *p* < 0.0001) and a significant effect of width on movement time (slope = −0.775 ± 1.409 × 10^−2^, mean ± SE, *p* < 0.0001) (Figure 4C), indicating that longer distances or smaller widths were associated with longer movement time. The interaction effect on movement time was non-significant between distance and width (*p* > 0.05). Throughput and percentage-of-overshoot are depicted in the Supplementary Figures S1, S2. The mean throughput across subjects was 5.741 ± 0.930 bit/s. Additionally, the percentage of overshoot showed that performance worsened with higher ID, which conforms to Fitts' Law.

**Figure 5:**
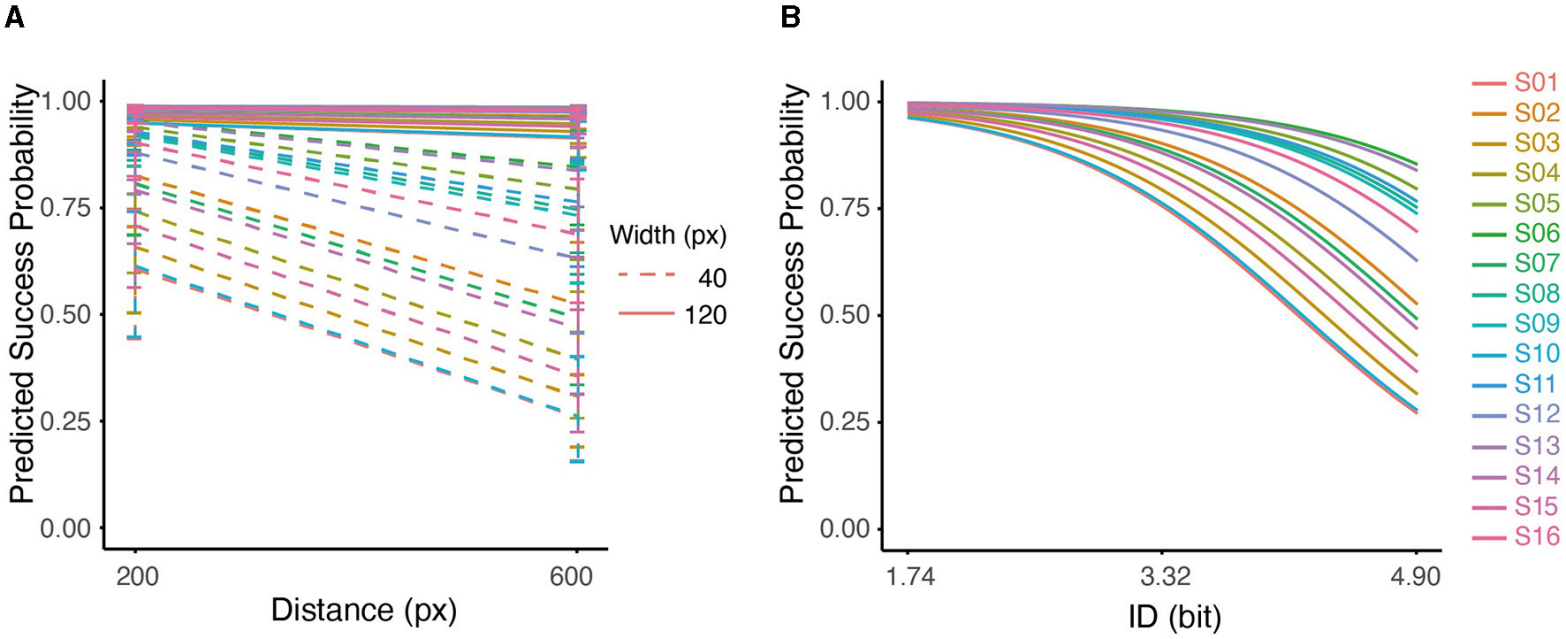
**(A)** Interaction between distance and width on predicted success probability for all subjects. **(B)** Predicted probability of success based on ID using Logistic Regression for all subjects. The effect of ID on predicted probability was significant (*p* < 0.001).

We examined the quality of task completion across all subjects by analyzing the binomial outcomes of success and failure (Supplementary Figure S3). GLMM analysis revealed a significant negative effect of distance (D) on the probability of success (Figure 5A), with a decrease of 0.4827/pixel (*p* < 0.001). Similarly, width (W) was found to have a significant positive effect on the probability of success, increasing by 2.7529/pixel (*p* < 0.001). It is worth noting that the interaction term (D × W) also had a significant impact on success (*p* < 0.05). Pair-wise comparisons showed that the probability of success was higher with shorter distance (D) only at a small width (W = 40px); at large width (W = 120px), the probability of success was not affected by D (*p* > 0.05). The conditional r^2^ value of the model was 0.5254, indicating that the fixed and random effects together explain approximately 53% of the variability in the response variable.

Furthermore, the GLMM analysis showed a significant effect of ID on the success outcome, with a decrease of 1.3407/bit (conditional r^2^ = 0.4893, *p* < 0.001). As can be seen in Figure 5B, the predicted probability of success dropped at higher IDs for all subjects. At higher IDs, the variability among participants also increased. This pattern indicated that at low IDs (1.74), participants accomplished the task with similar performances; As the ID increased (4.9), performances started to diverge, which perhaps indicated higher uncertainty in dealing with difficult tasks.

### 3.2 Contralateral activation analysis (channel-wise)

**Figure 6:**
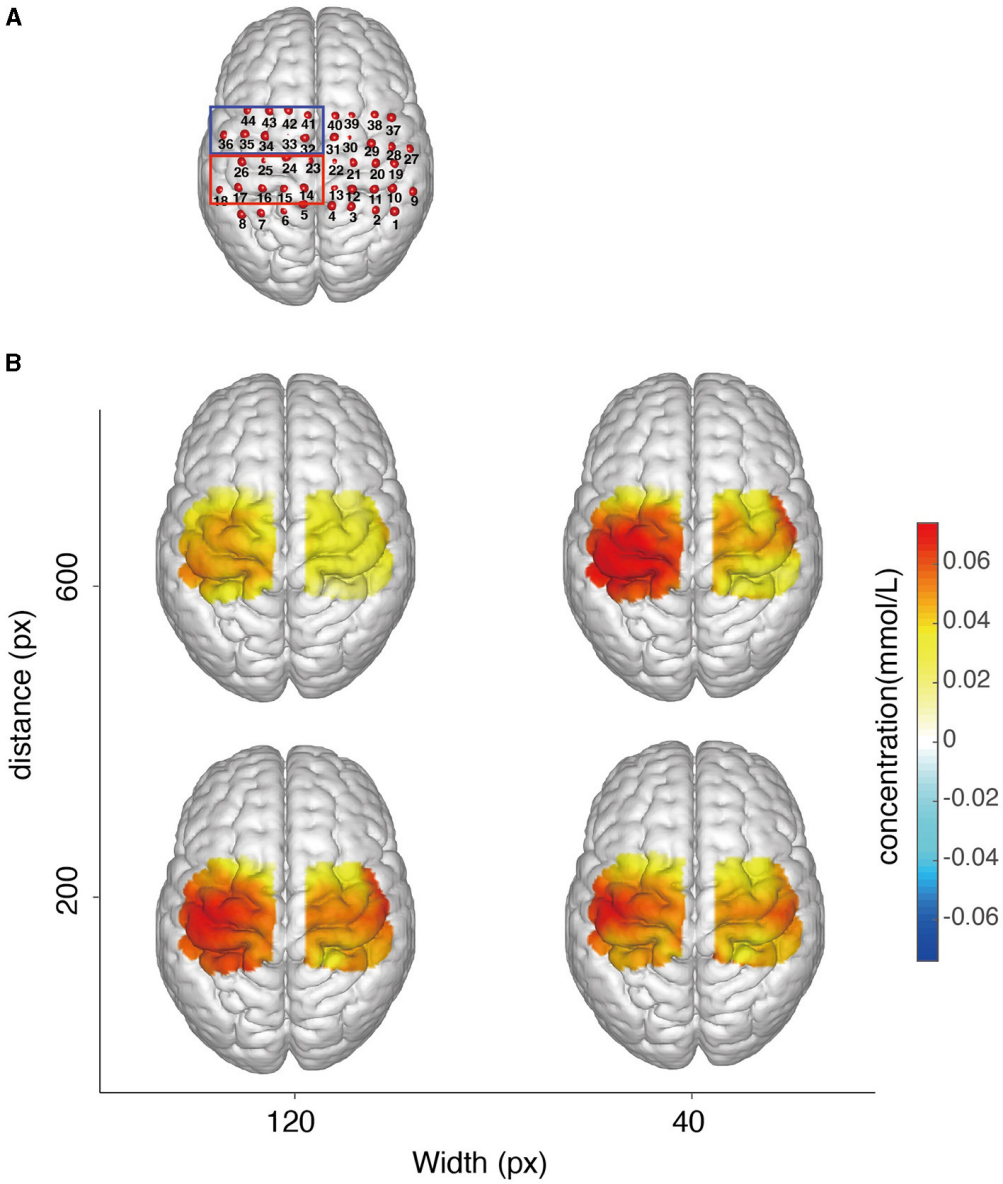
**(A)** Configurations of the three-dimensional location of each channel (S04). The spatial registration of each channel from real space to MNI coordinates is shown. Channels 14, 15, 16, 17, 18, 23, 24, 25, and 26 were categorized in the region of contralateral BA4 (red box) and channels 32, 33, 34, 35, 36, 41, 42, 43, and 44 were categorized in the region of contralateral BA6 based on the probabilistic registration (blue box). **(B)** Cortical activation under 4 conditions averaged across all participants.

We examined the MNI coordinates and the spatial registration of each channel. The 3D locations of all the channels are depicted in Figure 6A. The spatial registrations of channels in ROIs for a representative subject (S04) are listed in Table 1.

The map overview of activation in ROIs, interpolated from the averaged oxy-Hb concentration obtained by subtracting baseline values from those of the task phase, is presented in Figure 6B. Across all conditions, the contralateral BA4 and 6 showed activation, with greater activity observed in ROIs compared to their ipsilateral counterparts. Furthermore, the combinations of D200W120 and D600W40 exhibited a higher degree of ROI activation compared to all other conditions.

**Figure 7:**
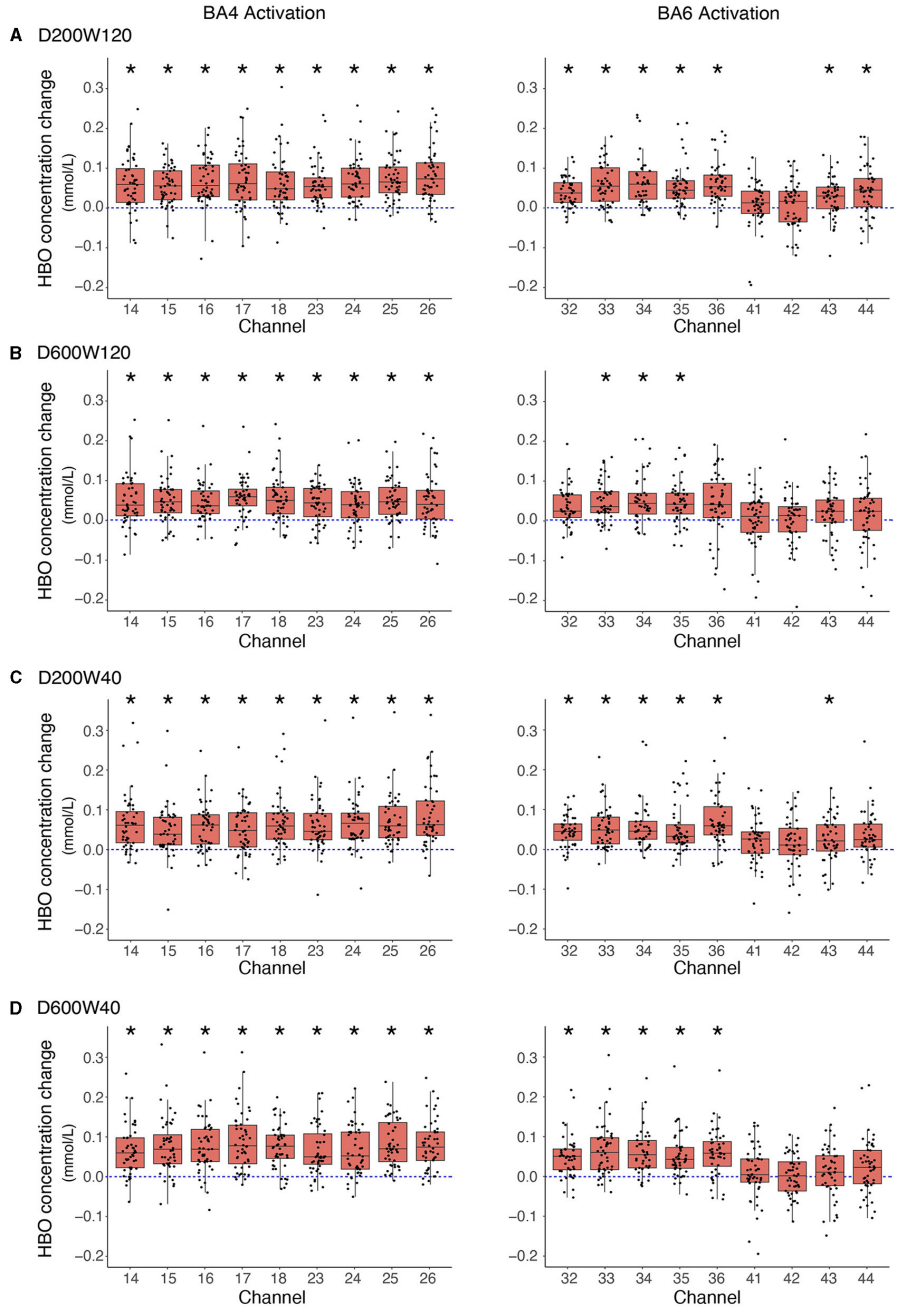
Group mean changes in Oxy-Hb responses for each task relative to the resting phase. Boxplot shows the median and percentile Oxy-Hb value of channels located in ROIs, and each scattered dot showing the Oxy-Hb value of each block. **(A)** The D200W120 condition. **(B)** The D600W120 condition. **(C)** The D200W40 condition. **(D)** The D600W40 condition. *Bonferroni-adjusted *p* < 0.05 from one-sample t-test.

In each condition, all channels corresponding to BA4 (channels 14, 15, 16, 17, 18, 23, 24, 25, and 26) in four conditions exhibited significant activation. In the D200W120 condition, significant activations were detected in 7 out of 9 BA6 channels (32, 33, 34, 35, 36, 43, 44, and all Bonferroni -adjusted *p* < 0.05). In the D600W120 condition, we discovered that only three BA6 channels (33, 34, 35, and all Bonferroni -adjusted *p* < 0.05) were significantly activated. In the D200W40 condition, six BA6 channels (32, 33, 34, 35, 36, 43, and all Bonferroni -adjusted *p* < 0.05) were activated. In the D600W40 condition, we found 5 channels in BA6 (32, 33, 34, 35, 36, and all Bonferroni -adjusted *p* < 0.05) significantly activated (Figure 7).

### 3.3 Contralateral activation analysis (ROI-wise)

**Figure 8:**
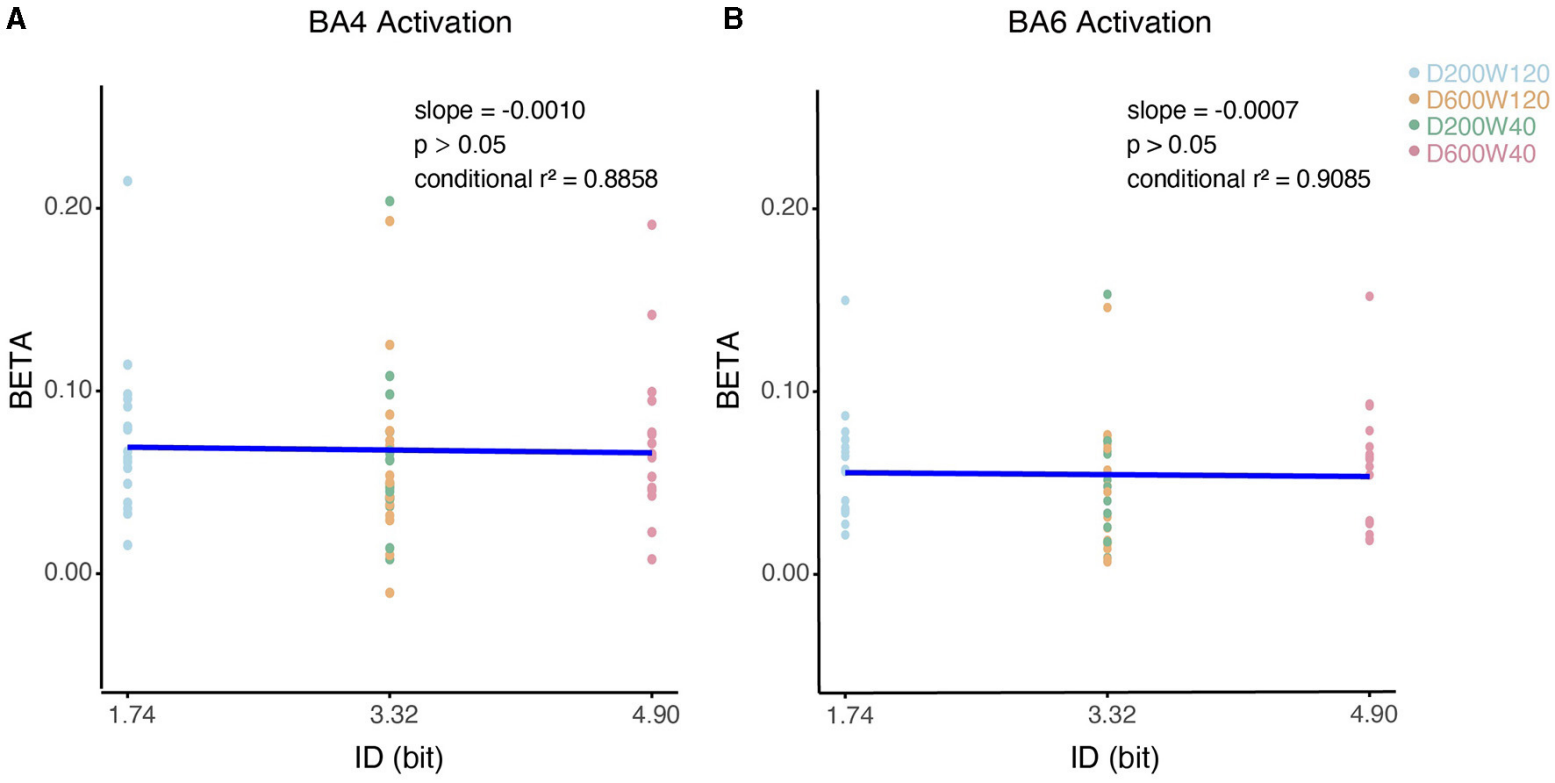
Contralateral relationship between ID and beta coefficients. **(A)** The impact of ID on the beta values for BA4 activation. **(B)** The impact of ID on the beta values for BA6 activation.

We analyzed the relationship between the activation of ROI (specifically BA4 and BA6) and ID using LMM (Figure 8), none of them showed significant correlation (*p* > 0.05).

**Figure 9:**
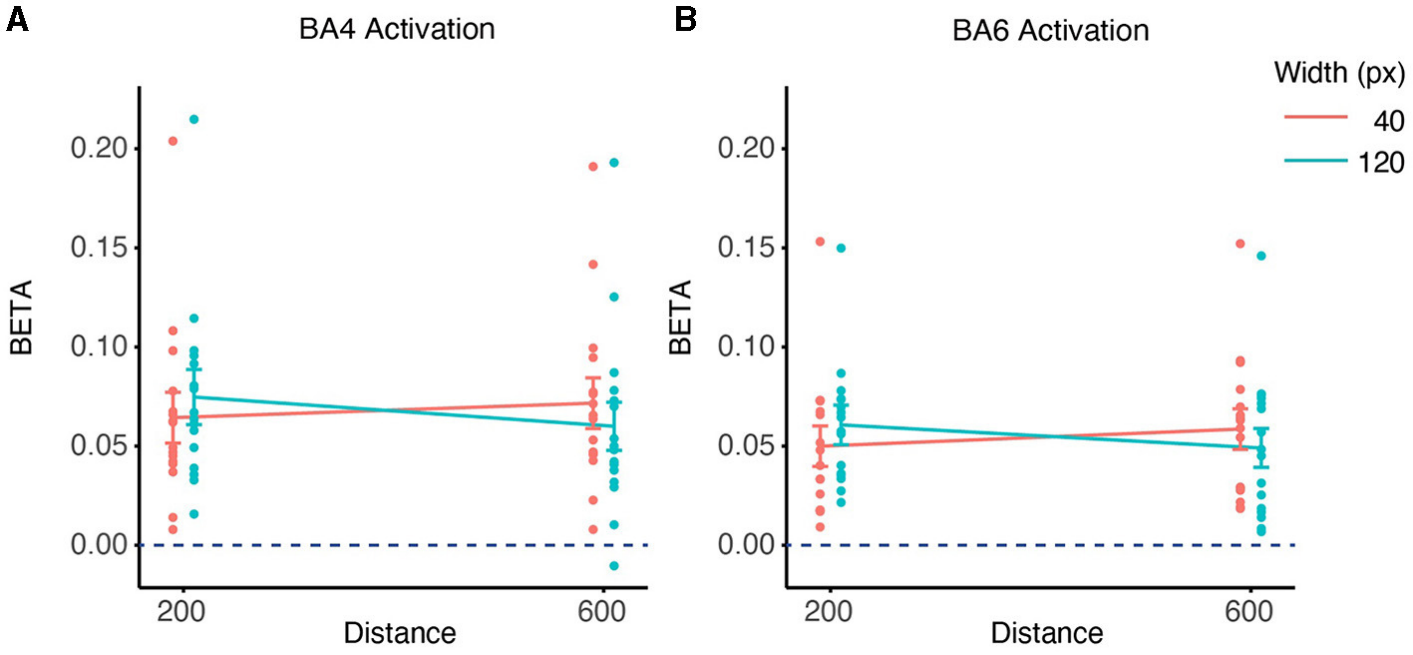
ROI-wise activation based on beta-values and interaction between distance and width on contralateral beta coefficients. **(A)** The activation of the BA4 region during the task was significant in the 4 conditions compared to the rest phase (*p* < 0.001). The interaction effects of distance and width on the beta values for BA4 activation were found to be significant (*p* < 0.05) based on a linear mixed effects model. **(B)** The activation of the BA6 region during the task was significant in the 4 conditions compared to the rest phase (*p* < 0.001). The interaction between distance and width was also significant in the beta values for BA6 activation (*p* < 0.001).

We did group analysis based on ROI based on Equation (2), significant activation was observed in both ROIs (BA4: W = 136, *p* < 0.001 BA6: W = 136, *p* < 0.001, Figures 9A, B). LMM analysis revealed significant differences for the effects of distance, width, and their interaction on the beta values for BA4 (Figure 9A) and BA6 (Figure 9B). There was a significant effect of Distance on BA4 activation (slope = 4.593 × 10^−5^ ± 2.021 × 10^−5^, mean ± SE, *p* < 0.05) and BA6 activation (slope = 4.684 × 10^−5^ ± 1.256 × 10^−5^, mean ± SE, *p* < 0.001), indicating that a longer distance was associated with higher activation in both BA4 and BA6.

There was also a significant effect of Width on BA4 activation (slope = 2.684 × 10^−4^ ± 1.010 × 10^−4^, mean ± SE, *p* < 0.05) and BA6 activation (slope = 2.606 × 10^−4^ ± 6.282 × 10^−5^, mean ± SE, *p* < 0.001), indicating that a wider target was associated with higher activation in both BA4 and BA6.

However, these effects were accompanied by a significant interaction between Distance and Width in BA4 activation (slope = −6.903 × 10^−7^ ± 2.259 × 10^−7^, mean ± SE, *p* < 0.01) and in BA6 activation (slope = −6.329 × 10^−7^ ±1.405 × 10^−7^, mean ± SE, *p* < 0.01). This means that the effect of distance/width on BA4 and BA6 activation varied depending on the level of width/distance. Specifically, distance had a stronger positive effect on BA4 and BA6 activation with a smaller width than with a larger width, whereas width had a similar positive effect on BA4 and BA6 activation for closer distances.

### 3.4 Ipsilateral activation analysis (ROI-wise)

Due to the role of ipsilateral (contralesional) hemisphere in stroke recovery (Buetefisch, 2015), ipsilateral activations were analyzed in channels 9, 10, 11, 12, 13, 19, 20, 21, and 22 (ipsilateral BA4) and channels 27, 28, 29, 30, 31, 37, 38, 39, and 40 (ipsilateral BA6). The Wilcoxon Rank Sum test on ROI-adjusted Beta (Equation 2) showed significant activations in both ROIs (BA4: W = 136, *p* < 0.001; BA6: W = 136, *p* < 0.001).

Further analyses on the ipsilateral ROI (Equations 6, 8) showed a positive correlation between Width and the Beta of BA4 (slope = 1.973 × 10^−4^ ± 7.722 × 10^−5^, mean ± SE, *p* < 0.05), indicating that a wider target was associated with higher activation in BA4. The interaction between D and W in BA4 was also significant (slope = −5.390 × 10^−7^ ± 1.727 × 10^−7^, mean ± SE, *p* < 0.01), indicating that distance had a stronger positive effect on BA4 activation with a smaller width than with a larger width; whereas width had a similar positive effect on BA4 activation for closer distances. None of the other correlations between Beta and task parameters (ID, Width, and Distance) were significant.

### 3.5 Handling of outliers

As can be seen in Figures 8, 9, subject S15 showed significantly higher Oxy-Hb level than others (higher than 150% the interquartile range of the third quartile). When data from S15 were excluded from the analysis, it neither changed our findings about the effect of Distance on BA6 activation, nor the effects of Width affected on BA4/6 activations. Nevertheless, the effect of Distance on BA4 activation became non-significant (*p* > 0.05) after removal of S15.

## 4 Discussion

In this study, we investigated the relationship between motor cortical activity and the variables of distance, width, and index-of-difficulty (ID) in upper-limb reaching movements within a horizontal plane. Sixteen healthy subjects performed reaching tasks with three levels of difficulty according to Fitts' Law. The motor performance and activity in the bilateral BA4 and BA6 areas were monitored. All subjects' reaching movements adhered to Fitts' Law, with an increase in index-of-difficulty resulting in a decrease of 1.3407/bit in success rate. Both contralateral BA4 and BA6 areas were activated across all conditions (*p* < 0.001). Notably, both BA4 and BA6 activations were stronger with longer distance (D) only at the small width (W = 40px); at large width (W = 120px), the trend was reversed. Overall, our data suggest that even though the motor performances conformed well with Fitts' Law, the activity in motor-related cortices cannot be parsimoniously explained by the index-of-difficulty.

One explanation for our primary finding—that ID is proportional with movement time but not cortical activation in BA4 and BA6—lies perhaps in the muscle activation necessitated by each combination of distance and target-width (Gottlieb, 1998). If the ID was increased via longer distances, the activation of BA4 and BA6 correlates with intensified activation of various upper limb muscles such as the brachioradialis and biceps brachii (Siemionow et al., 2000). As a contrast, if the ID was increased via smaller target-size it usually resulted in slower movement velocities (Corcos et al., 1989), which subsequently diminish muscle activation in the shoulder and elbow (Buneo et al., 1994). It is also noteworthy that in horizontal reaching movements, those over larger distances may traverse different muscle synergy zones (Gottlieb, 1998). For instance, a reach from the far left to the far right may entail an elbow movement from flexion to extension in our study, whereas movements with short distance may involve only elbow extension. Additionally, our reaching paradigm involves large-extent whole-arm movements, which is different from the pointing movements used in many studies (Winstein et al., 1997; Seidler et al., 2004; Buetefisch et al., 2014; Barany et al., 2020; Revill et al., 2022) and this would potentially explain the differences in cortical activation. Therefore, when reasoning about the motor-related cortical activation, it is insufficient to focus only on ID since it ignores the subtlety with muscle activation and movement paradigm.

The subjective perception of motor cost may also affect how individuals performed reaching in our experiments. Although the favored movement trajectories tend to minimize the motor cost associated with reaching (Harris and Wolpert, 1998), the subjective perception of effort may affect the motor cost and eventually the motor control (Steele, 2020). In principle, movements across longer distances are typically deemed more strenuous, but farther movements are not necessarily perceived more effort-taking (Morel et al., 2017). In our study, the success rates across different distances were close when the target-width remained large (Figure 5A, conditions D200W120 and D600W120).

Despite that conditions D200W40 and D600W120 had the same ID, these two conditions differed in success rate and cortical activation. As can be seen, D200W40 showed fewer trials of success (Supplementary Figure S3) and lower predicted success probability (Figure 5A); the cortical activation in this condition was also higher (Figure 6), which suggested that shorter movements had a higher variability, slower movement, and higher rates of error compared to larger distance movements (Borish et al., 2020). Shorter movements also might activate a cortical-subcortical loop involving the contralateral motor cortex, intraparietal sulcus, and caudate (Winstein et al., 1997). These results implied that combination of short distance and small target is more challenging. Ipsilateral activation seemed similar to that in the contralateral areas, i.e., BA4 and BA6 were activated in both hemispheres and all conditions. However, the interaction of D and W in the ipsilateral area were only observed in BA4, not in BA6. This indicated that reaching movement could cause cortical activation on both sides (Bundy and Leuthardt, 2019), but BA6 is less sensitive to the movement details in terms of ipsilateral activation.

Although we had estimated the sample size with an expected statistical power of 80%, the actual power of Beta_BA4 ~ Distance was below expectation (66%). This was also confirmed by the fact that “Beta_BA4 ~ Distance” turned non-significant after removal of S15. In regard to subject S15 who appeared an outlier in statistics, the data might still be valuable to include because this participant showed higher brain activation across all conditions, which may suggest increased cortical response due to elevated mental concentration (Choo et al., 2019). In our experiment, physiological and/or anatomical noises (Heinzel et al., 2013) might both yield inter-subject variability. These two types of noises cannot be distinguished using existing protocol and should be considered in future experiments.

During each trial of reaching, we imposed a timeout constraint of 2 seconds. The timing constraints appear critical for eliciting noticeable activation in motor-related cortical areas (van Mier et al., 1998; Churchland et al., 2006), yet the specific implications of such constraints impel further exploration. Although the main effect in “Beta ~ duration” was non-significant, this study did not control the block duration, which might directly affect hemodynamic responses (Khan et al., 2020). To clear the confounding from block duration, future protocols need to consider the repetition of movements, as well as a potential plateau effect for blocks longer than 20 seconds (Afzal Khan and Hong, 2021).

Several female candidates were excluded during the recruitment because of the poor fNIRS signal quality potentially due to thick hair. In future experiments, techniques such as individualized cap or improved hair-splitter may be used to ensure balanced gender ratio in recruitment. Participants in this study were healthy individuals between 50–65 years old, a demographic group at higher risk for neurodegenerative diseases (Kissela et al., 2012). Considering that aging is associated with less lateralized task-related activation of the primary motor cortices (Talelli et al., 2008), future studies may adjust the age range to align with clinical rehabilitation needs and the epidemiological profile of the patient population.

In clinic, if the training incorporated large-extent movements, our results suggested that activation in BA4 and BA6 might reflect how the participant respond to task difficulty, but the selection of Distance and Width still required further clarification due to their interacted effects on cortical activation.

## Data availability statement

The original contributions presented in the study are included in the article/Supplementary material, further inquiries can be directed to the corresponding authors.

## Ethics statement

The studies involving humans were approved by the Ethics Committee of Ruijin Hospital, School of Medicine, Shanghai Jiao Tong University (No. 209 of 2020). The studies were conducted in accordance with the local legislation and institutional requirements. The participants provided their written informed consent to participate in this study.

## Author contributions

HJ: Conceptualization, Data curation, Formal analysis, Investigation, Methodology, Software, Validation, Visualization, Writing – original draft, Writing – review & editing. ZC: Formal analysis, Investigation, Methodology, Writing – review & editing. YQ: Methodology, Writing – original draft. JY: Visualization, Writing – original draft. GC: Writing – original draft. QL: Writing – review & editing. LC: Resources, Writing – review & editing. YZ: Resources, Writing – review & editing. QX: Funding acquisition, Resources, Writing – review & editing. CN: Conceptualization, Funding acquisition, Project administration, Resources, Supervision, Writing – review & editing.

## Footnotes

^1^Package “emmeans.” Available online at: https://cran.r-project.org/web/packages/emmeans/emmeans.pdf (accessed June 8, 2024).
