# Supplementary material for: Hemodynamic activity is not parsimoniously tuned to index-of-difficulty in movement with dual requirements on speed-accuracy
Source: Front Hum Neurosci. 2024 Jul 9;18:1398601. doi: 10.3389/fnhum.2024.1398601 (PMC11263286; doi:10.3389/fnhum.2024.1398601)

Supplementary Material

# Supplementary Tables

# Table. S1 The MNI coordinates for 3 highest channels for S01.

| Channel | Anatomical Label in BA | MNI Coordinate | Probability |
| --- | --- | --- | --- |
| 26 | 4—Primary Motor Cortex | (-48.333, -16.667,61.333) | 0.551 |
| 14 | 4—Primary Motor Cortex | (-13.333, -37.667,80) | 0.508 |
| 25 | 4—Primary Motor Cortex | (-36.667, -17.333,72) | 0.493 |
| 32 | 6—Pre-Motor and Supplementary Motor Cortex | (-12.667, -2.333,75.667) | 1 |
| 33 | 6—Pre-Motor and Supplementary Motor Cortex | (-25.333, -1.333,71.667) | 1 |
| 34 | 6—Pre-Motor and Supplementary Motor Cortex | (-37,0,65) | 0.985 |

**Table. S2** **The MNI coordinates for 3 highest channels for S02.**

| Channel | Anatomical Label in BA | MNI Coordinate | Probability |
| --- | --- | --- | --- |
| 14 | 4—Primary Motor Cortex | (-7, -36,79) | 0.708 |
| 26 | 4—Primary Motor Cortex | (-45.667, -19.333,65) | 0.604 |
| 15 | 4—Primary Motor Cortex | (-18.333, -35.667,77.667) | 0.561 |
| 32 | 6—Pre-Motor and Supplementary Motor Cortex | (-9.333, -3.333,76) | 1 |
| 33 | 6—Pre-Motor and Supplementary Motor Cortex | (-19.333, -2.333,74) | 1 |
| 41 | 6—Pre-Motor and Supplementary Motor Cortex | (-7.333,11.667,72) | 0.996 |

**Table. S3** **The MNI coordinates for 3 highest channels for S03.**

| Channel | Anatomical Label in BA | MNI Coordinate | Probability |
| --- | --- | --- | --- |
| 23 | 4—Primary Motor Cortex | (-10.667, -20.667,79) | 0.611 |
| 25 | 4—Primary Motor Cortex | (-33.333, -21.667,74) | 0.601 |
| 14 | 4—Primary Motor Cortex | (-11.333, -37.333,80) | 0.554 |
| 32 | 6—Pre-Motor and Supplementary Motor Cortex | (-9.667, -5.333,77) | 1 |
| 33 | 6—Pre-Motor and Supplementary Motor Cortex | (-19.667, -5.333,75) | 1 |
| 34 | 6—Pre-Motor and Supplementary Motor Cortex | (-32.333, -4.333,67.333) | 1 |

**Table. S4** **The MNI coordinates for 3 highest channels for S04.**

| Channel | Anatomical Label in BA | MNI Coordinate | Probability |
| --- | --- | --- | --- |
| 14 | 4—Primary Motor Cortex | (-6.333, -35.333,79) | 0.756 |
| 26 | 4—Primary Motor Cortex | (-43.333, -19.333,66.667) | 0.642 |
| 15 | 4—Primary Motor Cortex | (-17.667, -35.667,78) | 0.566 |
| 32 | 6—Pre-Motor and Supplementary Motor Cortex | (-5.667, -4.667,75.333) | 1 |
| 33 | 6—Pre-Motor and Supplementary Motor Cortex | (-15.667, -3.333,75.333) | 1 |
| 34 | 6—Pre-Motor and Supplementary Motor Cortex | (-29.333, -3.667,69.333) | 1 |

**Table. S5** **The MNI coordinates for 3 highest channels for S05.**

| Channel | Anatomical Label in BA | MNI Coordinate | Probability |
| --- | --- | --- | --- |
| 23 | 4—Primary Motor Cortex | (-13.667, -25.667,79) | 0.853 |
| 35 | 4—Primary Motor Cortex | (-48.667, -13.333,59.667) | 0.498 |
| 36 | 4—Primary Motor Cortex | (-58.667, -15.667,50.333) | 0.317 |
| 33 | 6—Pre-Motor and Supplementary Motor Cortex | (-21.667, -10.333,75) | 1 |
| 41 | 6—Pre-Motor and Supplementary Motor Cortex | (-11.667,3.333,74) | 1 |
| 32 | 6—Pre-Motor and Supplementary Motor Cortex | (-12.667, -9.667,78) | 0.987 |

**Table. S6** **The MNI coordinates for 3 highest channels for S06.**

| Channel | Anatomical Label in BA | MNI Coordinate | Probability |
| --- | --- | --- | --- |
| 14 | 4—Primary Motor Cortex | (-9.333, -33.667,80) | 0.79 |
| 15 | 4—Primary Motor Cortex | (-19.333, -32.667,77) | 0.736 |
| 16 | 4—Primary Motor Cortex | (-33.333, -30.333,73) | 0.691 |
| 32 | 6—Pre-Motor and Supplementary Motor Cortex | (-9.333, -0.667,75) | 1 |
| 33 | 6—Pre-Motor and Supplementary Motor Cortex | (-16.667,1.333,74) | 1 |
| 41 | 6—Pre-Motor and Supplementary Motor Cortex | (-8.667,12.333,72) | 0.975 |

**Table. S7** **The MNI coordinates for 3 highest channels for S07.**

| Channel | Anatomical Label in BA | MNI Coordinate | Probability |
| --- | --- | --- | --- |
| 14 | 4—Primary Motor Cortex | (10.667, -28.667,80) | 0.987 |
| 16 | 4—Primary Motor Cortex | (-37.667, -26.667,71) | 0.711 |
| 15 | 4—Primary Motor Cortex | (-25.333, -25.667,75) | 0.706 |
| 24 | 6—Pre-Motor and Supplementary Motor Cortex | (-23.667, -10.667,74.333) | 1 |
| 32 | 6—Pre-Motor and Supplementary Motor Cortex | (-9.333,6.667,73.667) | 1 |
| 23 | 6—Pre-Motor and Supplementary Motor Cortex | (-10.667, -11.333,78) | 0.922 |

**Table. S8** **The MNI coordinates for 3 highest channels for S08.**

| Channel | Anatomical Label in BA | MNI Coordinate | Probability |
| --- | --- | --- | --- |
| 26 | 4—Primary Motor Cortex | (-46.667, -18.333,63.667) | 0.601 |
| 14 | 4—Primary Motor Cortex | (-10.333, -37.333,80) | 0.564 |
| 23 | 4—Primary Motor Cortex | (-10.667, -19.333,79) | 0.534 |
| 33 | 6—Pre-Motor and Supplementary Motor Cortex | (-18.667, -2.333,74) | 1 |
| 34 | 6—Pre-Motor and Supplementary Motor Cortex | (-32.667, -1.667,67) | 0.996 |
| 35 | 6—Pre-Motor and Supplementary Motor Cortex | (-46.667, -0.667,57.667) | 0.956 |

**Table. S9** **The MNI coordinates for 3 highest channels for S09.**

| Channel | Anatomical Label in BA | MNI Coordinate | Probability |
| --- | --- | --- | --- |
| 14 | 4—Primary Motor Cortex | (-10.333, -36.333,80) | 0.627 |
| 23 | 4—Primary Motor Cortex | (-10.667, -20.333,79) | 0.591 |
| 26 | 4—Primary Motor Cortex | (-45.667, -22.333,66) | 0.504 |
| 32 | 6—Pre-Motor and Supplementary Motor Cortex | (9.667, -4.333,76.333) | 1 |
| 33 | 6—Pre-Motor and Supplementary Motor Cortex | (-18.333, -4.333,75) | 1 |
| 34 | 6—Pre-Motor and Supplementary Motor Cortex | (-31.333, -4.667,68) | 1 |

**Table. S10** **The MNI coordinates for 3 highest channels for S10.**

| Channel | Anatomical Label in BA | MNI Coordinate | Probability |
| --- | --- | --- | --- |
| 14 | 4—Primary Motor Cortex | (-9.667, -34.333,80) | 0.743 |
| 26 | 4—Primary Motor Cortex | (-43.333, -17.667,66.667) | 0.646 |
| 15 | 4—Primary Motor Cortex | (-18.333, -35.333,77.667) | 0.585 |
| 32 | 6—Pre-Motor and Supplementary Motor Cortex | (-10.333, -2.667,76) | 1 |
| 33 | 6—Pre-Motor and Supplementary Motor Cortex | (-19.333, -2.333,74) | 1 |
| 35 | 6—Pre-Motor and Supplementary Motor Cortex | (-43.667, -0.667,60.333) | 0.96 |

**Table. S11** **The MNI coordinates for 3 highest channels for S11.**

| Channel | Anatomical Label in BA | MNI Coordinate | Probability |
| --- | --- | --- | --- |
| 14 | 4—Primary Motor Cortex | (-9.333, -31.667,80) | 0.887 |
| 15 | 4—Primary Motor Cortex | (-18.667, -30.667,77.333) | 0.821 |
| 16 | 4—Primary Motor Cortex | (-34.667, -29.333,72.333) | 0.699 |
| 32 | 6—Pre-Motor and Supplementary Motor Cortex | (-9.667, -0.333,75) | 1 |
| 33 | 6—Pre-Motor and Supplementary Motor Cortex | (-19.667,1.667,73) | 1 |
| 35 | 6—Pre-Motor and Supplementary Motor Cortex | (-44.333,3.333,59) | 0.988 |

**Table. S12** **The MNI coordinates for 3 highest channels for S12.**

| Channel | Anatomical Label in BA | MNI Coordinate | Probability |
| --- | --- | --- | --- |
| 14 | 4—Primary Motor Cortex | (-7.667, -30.667,79.333) | 0.943 |
| 15 | 4—Primary Motor Cortex | (-19.333, -27.667,77) | 0.835 |
| 16 | 4—Primary Motor Cortex | (-32.333, -28.333,73) | 0.74 |
| 24 | 6—Pre-Motor and Supplementary Motor Cortex | (-19.667, -9.333,76) | 1 |
| 32 | 6—Pre-Motor and Supplementary Motor Cortex | (-10,8,73) | 1 |
| 23 | 6—Pre-Motor and Supplementary Motor Cortex | (-9.667, -9.333,77.667) | 0.993 |

**Table. S13** **The MNI coordinates for 3 highest channels for S13.**

| Channel | Anatomical Label in BA | MNI Coordinate | Probability |
| --- | --- | --- | --- |
| 14 | 4—Primary Motor Cortex | (-9.667, -35.333,80) | 0.688 |
| 23 | 4—Primary Motor Cortex | (-10.333, -20.667,79) | 0.614 |
| 15 | 4—Primary Motor Cortex | (-19.667, -35.333,77) | 0.57 |
| 32 | 6—Pre-Motor and Supplementary Motor Cortex | (-8.667, -3.667,76) | 1 |
| 33 | 6—Pre-Motor and Supplementary Motor Cortex | (-18.333, -2.667,74.333) | 1 |
| 41 | 6—Pre-Motor and Supplementary Motor Cortex | (-10,10.333,72.667) | 0.997 |

**Table. S14** **The MNI coordinates for 3 highest channels for S14.**

| Channel | Anatomical Label in BA | MNI Coordinate | Probability |
| --- | --- | --- | --- |
| 25 | 4—Primary Motor Cortex | (-37.333, -19.333,71.667) | 0.615 |
| 14 | 4—Primary Motor Cortex | (-11.667, -36.333,80) | 0.607 |
| 15 | 4—Primary Motor Cortex | (-23.333, -36.333,76) | 0.474 |
| 32 | 6—Pre-Motor and Supplementary Motor Cortex | (-10.333, -1.667,75.667) | 1 |
| 33 | 6—Pre-Motor and Supplementary Motor Cortex | (-18.667, -0.667,74) | 1 |
| 34 | 6—Pre-Motor and Supplementary Motor Cortex | (-34.333,0.333,66) | 0.968 |

**Table. S15** **The MNI coordinates for 3 highest channels for S15.**

| Channel | Anatomical Label in BA | MNI Coordinate | Probability |
| --- | --- | --- | --- |
| 23 | 4—Primary Motor Cortex | (-10.333, -21.333,79) | 0.647 |
| 26 | 4—Primary Motor Cortex | (-49.667, -14.667,59.333) | 0.485 |
| 25 | 4—Primary Motor Cortex | (-37, -16.667,71.333) | 0.474 |
| 32 | 6—Pre-Motor and Supplementary Motor Cortex | (-5.333, -6.333,75.333) | 1 |
| 33 | 6—Pre-Motor and Supplementary Motor Cortex | (-17.667, -3.333,75) | 1 |
| 35 | 6—Pre-Motor and Supplementary Motor Cortex | (-44.667,2,59) | 0.968 |

**Table. S16** **The MNI coordinates for 3 highest channels for S16.**

| Channel | Anatomical Label in BA | MNI Coordinate | Probability |
| --- | --- | --- | --- |
| 14 | 4—Primary Motor Cortex | (-9.667, -33.333,80) | 0.799 |
| 15 | 4—Primary Motor Cortex | (-19.667, -32.667,77) | 0.732 |
| 26 | 4—Primary Motor Cortex | (-43.667, -18.667,66.333) | 0.647 |
| 32 | 6—Pre-Motor and Supplementary Motor Cortex | (-10.667, -3.333,76) | 1 |
| 33 | 6—Pre-Motor and Supplementary Motor Cortex | (-18, -2,74) | 1 |
| 41 | 6—Pre-Motor and Supplementary Motor Cortex | (-8.667,9.667,73) | 0.968 |

# Supplementary Figures

**Figure. S1** The Throughput for each subject in successful trials.


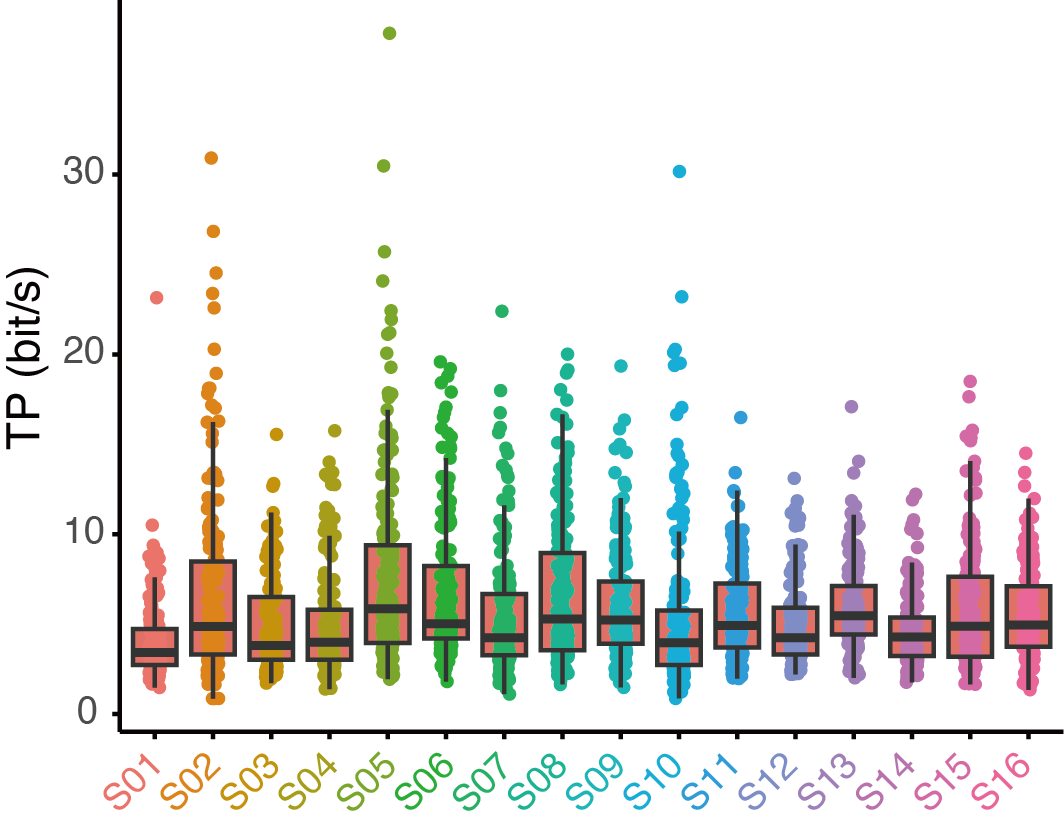


**Figure. S2** The percentage of overshoot for each individual across three distinct IDs.


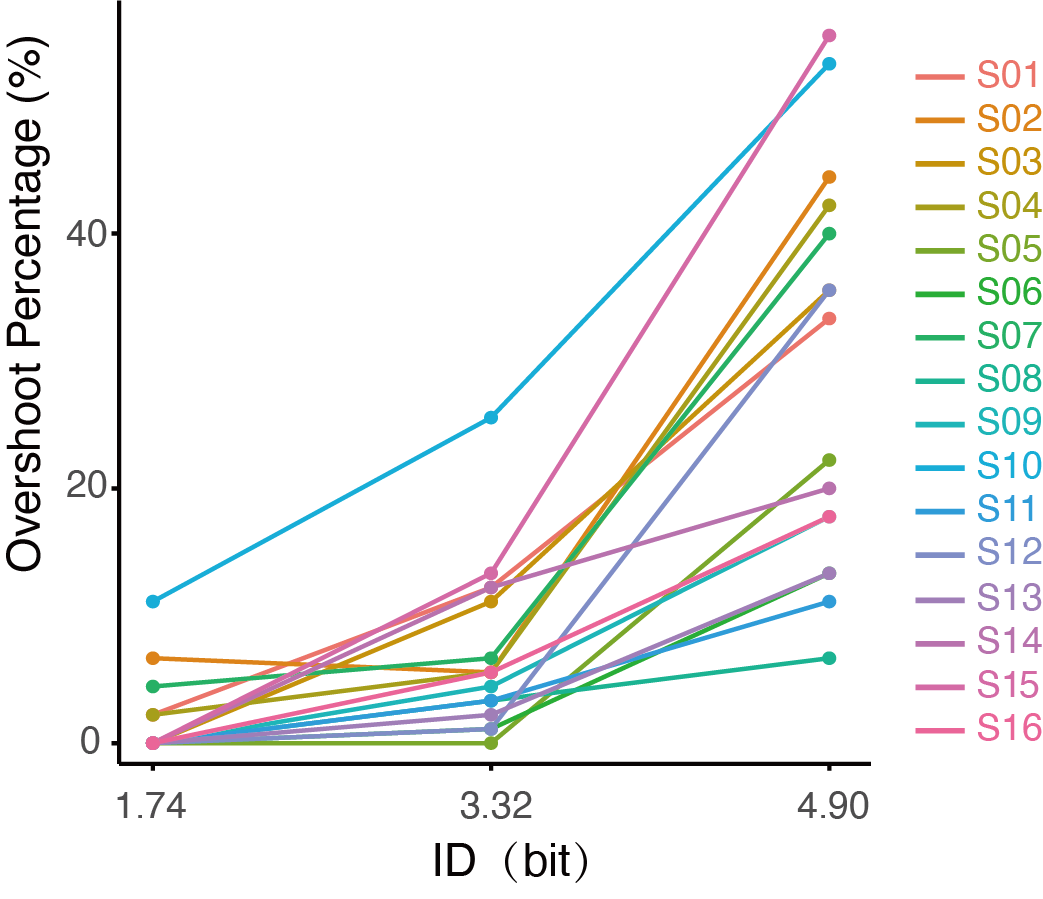


**Figure. S3** Binomial outcomes of success for 16 subjects. Each subject's outcomes across all trials re depicted by 45 markers, where a solid point indicates success, and an empty point indicates failure.


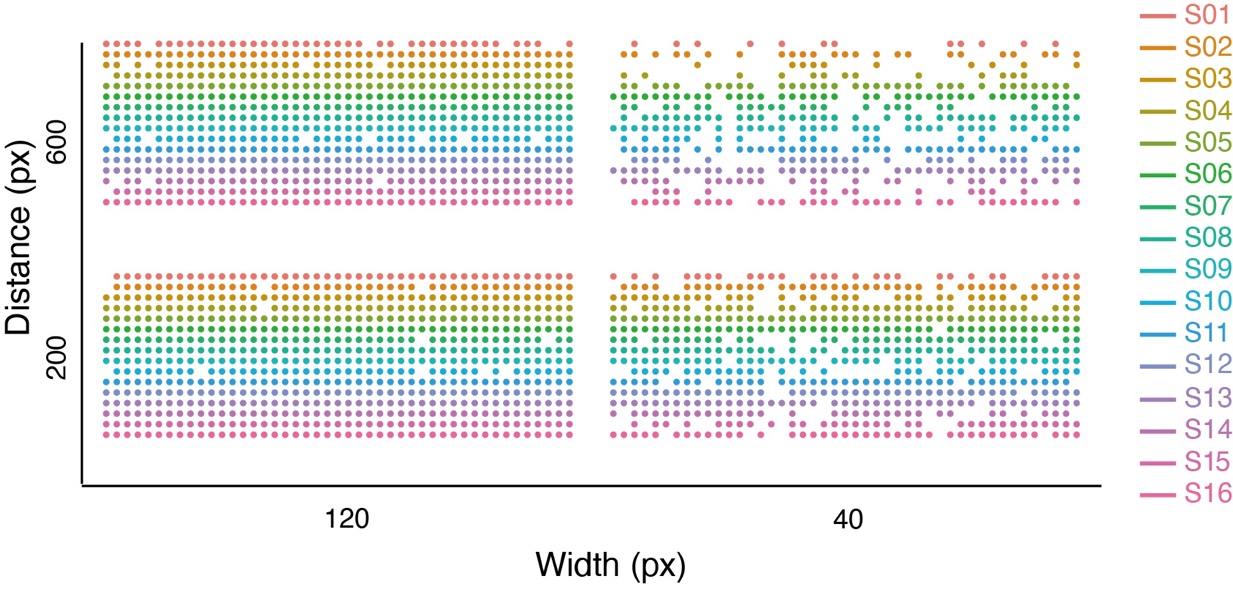

Supplement: Supplementary file 1 [file Data_Sheet_1.docx]
